# Identification of Phenolic Compounds in the Invasive Plants Staghorn Sumac and Himalayan Balsam: Impact of Time and Solvent on the Extraction of Phenolics and Extract Evaluation on Germination Inhibition

**DOI:** 10.3390/plants13233339

**Published:** 2024-11-28

**Authors:** Maja Mikulic-Petkovsek, Eva Ravnjak, Denis Rusjan

**Affiliations:** Department of Agronomy, Biotechnical Faculty, University of Ljubljana, Jamnikarjeva 101, SI-1000 Ljubljana, Slovenia; 12evamisic45@gmail.com (E.R.); denis.rusjan@bf.uni-lj.si (D.R.)

**Keywords:** *Rhus typhina* L., *Impatiens glandulifera* Royle, phytochemistry, phenolic components, extraction efficiency, invasive plant species, germination reduction

## Abstract

An HPLC-MS-DAD analysis of phenolic compounds was carried out on the extracts of staghorn sumac (*Rhus typhina* L.) and Himalayan balsam (*Impatiens glandulifera* Royle). This study focuses on the influence of solvent type and extraction time on the phenolic extraction efficiency from both invasive plants. Methanol extraction resulted in a 4.2 times higher content of hydroxybenzoic acids, a 3.7 times higher content of hydroxycinnamic acids, a 3.2 times higher content of flavanols, a 9.6 times higher content of flavanones, and an 8.7 times higher content of flavonols in the Himalayan balsam extract compared to aqueous extraction. Anthocyanins were only detected in the alcohol-based extraction. In comparison, the aqueous extraction from staghorn sumac resulted in a higher yield of total hydroxybenzoic acids, hydroxycinnamic acids, and flavonols, while the methanol extraction resulted in a 1.4-fold lower total polyphenolic content compared to the aqueous extraction. The type of solvent had no significant effect on the total content of flavanols in staghorn sumac. Extraction time considerably affected the total phenolic content in both plant extracts. After 84 h of extraction, the staghorn sumac extract showed a 3.5-fold increase in the total phenolic content compared to the initial measurement. In contrast, the Himalayan balsam extract showed a 40% decrease in the total phenolic content after 84 h. The Himalayan balsam extract reduced the germination of perennial ryegrass seeds by 55%, while the staghorn sumac extract reduced it by 80%. Both extracts also inhibited shoot and root growth of perennial ryegrass, although the Himalayan balsam extract at a concentration of 0.125 g/mL stimulated root growth of perennial ryegrass. The strategic use of invasive alien plants could be an effective approach to control their spread in the environment, potentially reducing management costs. The effectiveness of this approach depends largely on the type and content of allelochemicals present in the invasive plants.

## 1. Introduction

Invasive alien plants (IAP) are spreading all over the world. The speed and spread of their invasion depend on climate conditions and their adaptability. The increase in invasive alien species is a typical biological invasion that poses a growing threat to biodiversity, ecosystem change, and human health [1,2]. Various studies have shown that invasive alien plant species have strong ecological adaptability; they adapt by changing morphological and generative traits in order to survive, develop, and thrive better under new circumstances [3]. The strong ecological adaptability of invasive species is demonstrated by their high genetic variability, increased tolerance to environmental stressors, broad ecological range, and the ability to produce compounds that suppress the growth of other plant species [2]. There is evidence that allelopathy often plays an important role in the success of their invasion and has an impact on native plant species [1].

The term allelochemical or allelopathic compound refers to the role of the compound, but not to the actual chemical identity, since compounds in nature can have several roles depending on the organism, environment, and other influences acting on the organism [4]. The effect of allelochemical compounds is diverse and affects many biochemical reactions, leading to a change in various physiological functions. The results of allelochemical influences can, therefore, be observed at different levels of plant organization: molecular, structural, biochemical, physiological, and ecological [5].

Allelochemicals are classified as secondary plant substances. These secondary metabolites are typically produced by specialized cells or tissues in plants. They are often synthesized in response to unfavorable environmental conditions or during certain stages of development [4]. Allelopathic chemicals, particularly phenolic compounds, play a critical role in the success of invasive plant species by inhibiting the growth and germination of native plants. Invasive species release phenolic compounds, such as tannins, flavonoids, and lignins, into the environment to suppress competing plants. Phenolic compounds affect neighboring plants by interfering with nutrient uptake, altering hormone pathways, or directly inhibiting root and shoot development [6].

The allelopathic effect contributes to the aggressive spread of many invasive species, making them difficult to control, which poses a significant threat to global biodiversity [6]. The possibility of using invasive alien plants for various purposes has become an effective strategy for their control and management, which also aids in reducing the costs of preventing and controlling their spread. Their benefits depend on the type of plant and the stage and extent of its invasion process [2].

For this reason, investigating the phytochemical composition of invasive plant species and exploring their potential beneficial uses is crucial. The secondary metabolites of these species can be utilized in various ways, including pharmaceutical, agricultural, and industrial applications [7]. To effectively extract these bioactive compounds, it is essential to examine how different extraction factors affect their yield. Key parameters that require optimization include the type and concentration of solvent, extraction time, and temperature [8]. Secondary metabolites, including phenolic compounds, are sensitive to these conditions and demand optimized methods for efficient extraction [9].

The success of the extraction is most frequently determined with the following solvents: methanol, acetone, ethanol, and water. Ethanol is often the solvent of choice as it is efficient and environmentally friendly. For instance, aqueous ethanol solutions have been reported to enhance the solubility of phenolics and, consequently, increase extraction yields due to their polarity. Additionally, extraction duration plays an important role, with a longer duration generally increasing the phenolic content of the plant material. However, an extended duration also risks degrading certain compounds, especially at higher temperatures [10,11,12].

Several environmental issues have been associated with two invasive species—staghorn sumac and Himalayan balsam—which are spreading rapidly in various parts of the world, causing numerous problems due to their swift growth. Since the efficiency of constituent extraction from the plant parts of staghorn sumac (*Rhus typhina* L.) and Himalayan balsam (*Impatiens glandulifera* Royle) has not been researched much, this study set several objectives. Because there is little information on the content of phenolic compounds in staghorn sumac and Himalayan balsam in the literature, the primary aim (I) was to identify phenolic compounds and their content in the aerial parts of these two invasive plants. The second objective (II) was to determine which solvent achieves the most efficient extraction of phenolics from the invasive plants, and the third objective (III) was to assess the impact of extraction duration on the yield of phenolics. The fourth objective (IV) was to determine whether water extracts from these two plants could influence the reduction in seed germination of certain plant species. Parameters, such as seed germination percentage, shoot length, and root length, were monitored, as well. From a practical perspective, the study sought to determine whether extracts of these two invasive plant species could potentially be used as a weed-control method in agriculture.

## 2. Results and Discussion

### 2.1. Identification of Phenolic Compounds and Extraction Efficiency of Phenolics from Staghorn Sumac

Forty-eight phenolic substances from four phenolic groups were detected in the staghorn sumac extract. Detailed information on the identified phenolic compounds and HPLC-MSn fragmentation data in the staghorn sumac extract is provided in Supplementary Table S1, with chromatograms displayed in Figure S1. Flavonols were the most prominent phenolic groups, as they accounted for 63% of the total analyzed phenolic content (TAP) analyzed in both the alcoholic extract (16,842 µg/g DW) and the aqueous extract (22,628 µg/g DW) (Table 1). The predominant flavonols were myricetin-3-rhamnoside and quercetin-3-rhamnoside, which together accounted for 60% of the total flavonol content. In addition, four kaempferol derivatives, seven quercetin derivatives, five myricetin derivatives, and one isorhamnetin were identified. Earlier studies also detected many flavonols in staghorn sumac [13,14]. Flavanols were the second most prevalent phenolic group, accounting for 15% of the total phenolics in the aqueous staghorn sumac extract and 26% in the alcoholic extract. From the flavanol group, only catechin and various procyanidins were identified. The group of hydroxycinnamic acids derivatives had a relatively low content, although having quite a large number of analyzed representatives (Table 1). HCA accounted for only 2% of the TAP content. Amongst the phenolic acids, hydroxybenzoic acids were also determined, and their content in the staghorn sumac aqueous extract was 2369 µg/g DW. Liu et al. [13] and Olchowik et al. [14] also determined derivatives of hydroxybenzoic acids. Moreover, Katiki et al. [15] found derivatives of ellagic acid in the leaves of *Rhus typhina,* and Liu et al. [13] identified the flavanone fustin in the stems of the same species, none of which were determined in this experiment.

To achieve precise extraction of diverse phenolic compounds from plants, it is essential to use a range of solvents with varying polarities [16]. When comparing the effectiveness of solvents in extracting higher levels of phenolic compounds, it was observed that using water resulted in a 3-fold increase in hydroxybenzoic acids, a 1.4-fold increase in HCA, and a 1.34-fold increase in flavonol content. Aside from the total flavonol content, there were no significant differences observed between the two solvents. In previous scientific studies, water also proved to be a better solvent for the extraction of polyphenolics from green walnut shells (*Juglans regia*) [17] and papaya leaves (*Carica papaya*) [18]. On the other hand, alcohol proved to be a better solvent for the extraction of saponins and flavonoids from papaya leaves [18]. Martin et al. [19] reported contrasting findings; they found that the extraction yield of phenolic compounds from staghorn sumac (*Rhus typhina*) leaves was significantly higher when using water or 70% ethanol as the solvent compared to methanol. However, this trend was not observed for staghorn sumac berries, where no significant difference in extraction yield was noted between methanol and water.

### 2.2. Identification of Phenolic Compounds and Extraction Efficiency of Phenolics from Himalayan Balsam

All identified phenolic compounds and HPLC-MSn fragmentation data from the Himalayan balsam extract are presented in Supplementary Table S2, with chromatograms shown in Figure S2. In the Himalayan balsam extract, six representatives from the anthocyanin group, two hydroxybenzoic acids, seven hydroxycinnamic acids, four flavanols, and eleven flavonol glycosides (five kaempferol derivatives, four quercetin derivatives, one isorhamnetin derivative, and one myricetin derivative), and three representatives from the flavanone group were identified (Table 2).

In the Himalayan balsam flowers, Vieira et al. [20] also determined myricetin from the group of flavonols. The most dominant hydroxybenzoic acid was protocatechuic acid. Vanillic acid was also determined. Similarly, Szewczyk and Olech [21] found that gallic, gentisic acid, and syringic acids are all represented in the flowers. In the group of hydroxycinnamic acids, ferulic, *p*-coumaric, and caffeic acids were analyzed. In addition to the previously listed acids, Szewczyk and Olech [21] also found 3-hydroxycinnamic, salicylic, sinapic, and veratric acid, which were not found in the aerial parts of Himalayan balsam in this experiment. Two solvents (80% MeOH and 100% H_2_O) were used for the extraction of phenolic substances. The flavonol glycosides were extracted most efficiently with alcohol, accounting for 35% of all phenolics analyzed. These were followed by flavanols (24% of the total phenolic content, TAP) and flavanones (23% of TAP). The highest proportion of flavanols (42%) was extracted with water, followed by flavonols (23% of TAP). In addition, anthocyanins, including two cyanidin derivatives, two delphinidin, and two malvidin derivatives, were detected in the fruits of Himalayan balsam. The anthocyanins were extracted with alcohol only, as they could not be detected in the aqueous extract. Characteristically higher extraction efficiency of almost all phenolics (except kaempferol-3-rutinoside, quercetin-3-glucoside) was achieved with 80% methanol. The content of flavonols was 8.7 times higher in the metanolic extract than in the aqueous extract, the content of flavanols was 9.6 times higher, the content of HCA was 3.7 times higher, and the content of flavanols was 3.2 times higher in the alcoholic extract than in the aqueous extract. It should be emphasized that the alcoholic extract of Himalayan balsam had an extremely high content of isohramentin-3-rutinoside, eriodictyol hexoside 1, epicatechin, and quercetin-3-rutinoside. Similarly, Al-Muwaly et al. [22] found that the methanolic extract of *Rhus coriaria* fruit had the highest content of phenolic compounds and flavonoids, and the aqueous and ethanolic extracts had the lowest. One reason for the higher extraction yield observed with methanol is its high demonstrated efficacy in precipitating proteins [19]. However, it has also been reported that esterification reactions occurring in the presence of methanol can lead to the degradation of polyphenolic compounds, lipids, and saponins in extracts from the plant material [23].

Previous scientific research has shown that no generally recognized solvent is best suited for the extraction of a wide range of different polyphenolics. Nevertheless, solvents with higher polarity (e.g., methanol and ethanol) are often considered to provide the best conditions for optimal extraction of polyphenolics, mainly due to the high solubility of polyphenolics in these solvents [12]. On the other hand, water is the only naturally occurring extraction solvent providing more accurate data on the content of phenolic compounds in plant parts and their potential allelopathic activity in nature [24].

### 2.3. Effect of Aqueous Staghorn Sumac and Himalayan Balsam Extracts on Seed Germination and Plant Growth of Perennial Ryegrass

The effect of the aqueous extracts of Himalayan balsam and staghorn sumac on the germination of perennial ryegrass seeds, as well as the shoot and root lengths after treatment with the extract, is shown in Table 3. Both plant extracts exhibited inhibitory effects on seed germination. On average, the Himalayan balsam extract reduced seed germination by 26.3% and 55% compared to the control. The staghorn sumac extract was even more effective, reducing germination by 8182% compared to the control (Table 3).

Previous studies have reported that Himalayan balsam leaf extracts exhibit a germination-inhibitory effect on the seeds of white mustard [25], rapeseed, and wheat [26]. Similar results were observed in germination tests involving lettuce seeds. As the concentration of the staghorn sumac leaf extract increased, the percentage of germinated seeds decreased. However, at lower concentrations of the leaf extract used, the shoot biomass and vigor index increased [27]. The aqueous extract of staghorn sumac significantly reduced the growth of perennial ryegrass roots, with treated roots being 18.5–19.2 mm shorter than those of the control plants (Table 3). Interestingly, the water extract of Himalayan balsam did not cause a notable reduction in perennial ryegrass root growth. A low concentration of the Himalayan balsam extract even stimulated the growth of perennial ryegrass roots. At a concentration of 0.125 g/mL, the perennial ryegrass roots were, on average, 5.7 mm longer than those of the control (seeds irrigated with water) (Table 3).

Zhong et al. [28] reported that a water staghorn sumac extract reduced lettuce radicle length by 96% and leaf length by 27% compared to the control (seeds treated with water). This suggests that dense populations of staghorn sumac plants in an area produce a large mass of leaves. A staghorn sumac leaf extract, known for having an allelopathic effect, could also have a great impact on reducing native plant populations, thereby enhancing the invasive success of staghorn sumac.

The results of this experiment, along with previous studies, demonstrate that extracts from certain invasive plants can negatively impact the growth and development of selected plants tested [25,26,29,30]. It is assumed that the allelopathic effect of staghorn sumac is largely due to its high content of secondary metabolites, particularly terpenoids and phenolic compounds. Among the terpenoids, the major components of sumac essential oils are monoterpene and sesquiterpene hydrocarbons [31]. The predominant phenolic compounds include flavonoid derivatives and hydrolyzed tannins [32]. Extracts from invasive species have also been utilized in organic farming as natural fungicides. For instance, some essential oils, such as lemonene, α-pinene, and β-pinene, have been demonstrated to have an antifungal effect against the fungus *Botrytis cinerea* on strawberries [33]. According to Ganiee et al. [34], plant leaves generally contain a higher content of phenolic substances than roots, contributing to the strong allelopathic effect of leaf extracts. Previous research has also shown that the allelopathic effect of certain plants is mostly due to the synthesis of secondary metabolites [35,36]. The allelopathic effect of phenolic compounds is probably related to their ability to inhibit plant growth, non-specific changes in cell membrane permeability, and the alteration of the activity and function of certain enzymes [24].

### 2.4. Influence of Extraction Time on the Efficiency of Phenolic Compound Extraction from Staghorn Sumac and Himalayan Balsam

The third section of this study examines the impact of extraction duration, with a focus on identifying the optimal time for efficiently extracting specific phenolic compounds from the plant material of the two studied invasive species. The duration of extraction is a vital factor in solvent extraction experiments as it influences the mass transfer equilibrium of compounds into the extraction solvent [37]. From a practical point of view, the goal was to determine the optimum extraction time to maximize the secondary metabolites’ concentration in the aqueous extract. This assumption is based on the fact that higher concentrations enhance the extract’s inhibitory effect on plant germination and growth. The extraction temperature was maintained constant at 4 °C throughout the process.

**Figure 1:**
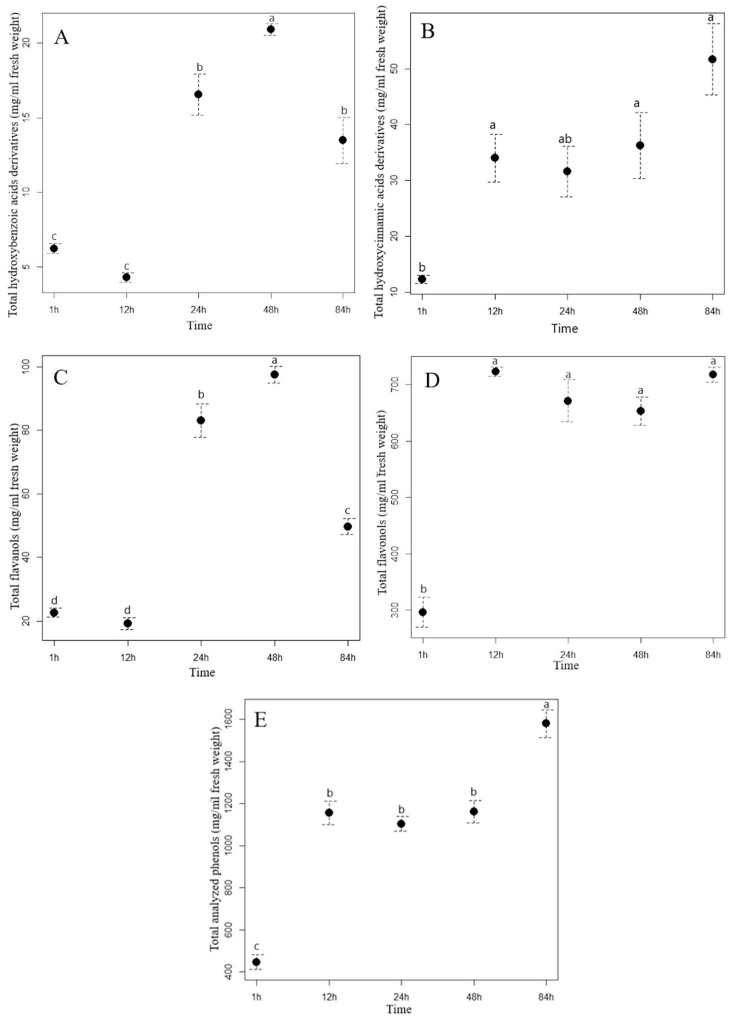
The contents (mean ± standard error in mg/mL) of total hydroxybenzoic acid derivatives (**A**), total hydroxycinnamic acid derivatives (**B**), flavanols (**C**), flavonols (**D**), and total analyzed phenolics (**E**) in the staghorn sumac water extract in different extraction time. Different letters indicate statistical differences in the content of the phenolic group between different extraction times (Tukey HSD test). (T1 = 1 h of the extraction, T2 = 12 h of the extraction, T3 = 24 h of the extraction, T4 = 48 h of the extraction, and T5 = 84 h of the extraction).

**Figure 2:**
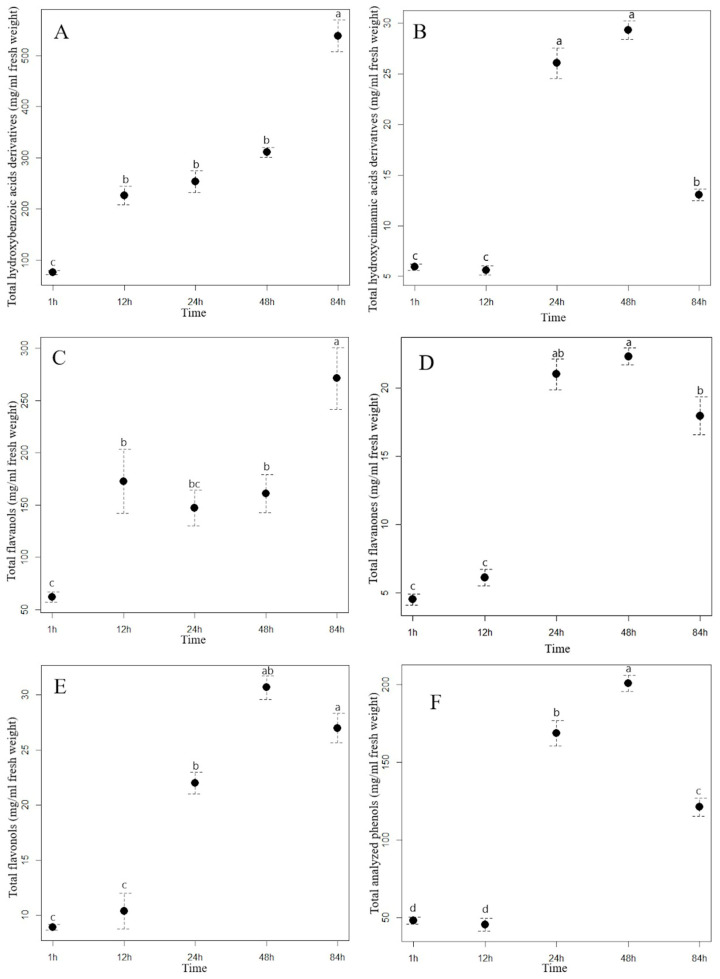
The contents (mean ± standard error in mg/mL) of total hydroxybenzoic acid derivatives (**A**), total hydroxycinnamic acid derivatives (**B**), flavanols (**C**), flavanones (**D**), flavonols (**E**), and total analyzed phenolics (**F**) in the Himalayan balsam water extract at different extraction times. Different letters indicate statistical differences in the content of the phenolic group between different extraction times (Tukey HSD test). (T1 = 1 h of the extraction, T2 = 12 h of the extraction, T3 = 24 h of the extraction, T4 = 48 h of the extraction, and T5 = 84 h of the extraction).

Extraction time had a different effect on the content of the analyzed hydroxybenzoic acids (HBA) in the aqueous extracts of staghorn sumac and Himalayan balsam. In the staghorn sumac extract, the content of total hydroxybenzoic acids increased consistently with a longer extraction time (Figure 1A, Supplementary Table S3). From the beginning of the experiment (1 hour after extraction) to the final time point (84 h), the acid content of the staghorn sumac extract increased by an average of 462.66 mg/mL, representing a sevenfold increase. Supplementary Table S3 shows that the contents of almost all HBA compounds in the staghorn sumac extract (except for galloylhexose 1) increased significantly at the final time point. The substantial increase in total HBA content over 84 h is primarily due to an 8.4-fold rise in gallic acid, which accounted for 85% of the total HBA content. In contrast, the Himalayan balsam extract reached its highest HBA content after 48 h (Figure 2A, Supplementary Table S4), with a 3.4-fold increase compared to the first time point (1 h after extraction, 6.24 mg/mL extract). Shi et al. [38] reported that water-based HBA extraction achieved maximum absorption capacity within the first 20 min, with the adsorption equilibrium or saturation point reached after 40 min. In certain non-aqueous solvents, optimal HBA extraction generally occurs within 15–30 min, when solute recovery increases [37]. Extending extraction time beyond half an hour may often result in HBA degradation due to oxidation.

The most important hydroxycinnamic acids in staghorn sumac were 3-caffeoylquinic and caffeic acids, which accounted for up to 64% of the total HCA content after one hour of water extraction. However, their proportion increased with increasing extraction time, as both phenolic acids accounted for up to 73% of the total HCA content after 84 h of extraction (Supplementary Table S3). A longer extraction time resulted in a significant increase in the total HCA content in the aqueous extract of staghorn sumac (Figure 1B, Supplementary Table S3), with a fourfold increase at T5 (84 h) compared to T1 (1 h). In the Himalayan balsam extract, the total HCA content substantially increased after 24 h of extraction (rising by 20 mg/mL compared to T1). However, a notable decrease occurred after 84 h, with the total HCA content being 16 mg/mL lower than at T4 (Figure 2B, Supplementary Table S4). At the end of the experiment, the average content of the total hydroxycinnamic acids measured in the extract of Himalayan balsam was 2.25-fold lower than the maximum average content of these acids measured after 48 h of extraction. Vergara-Salinas et al. [39] obtained similar results upon HCA extracting from thyme; the content decreased significantly with increasing extraction time.

The content of total flavanols analyzed changed significantly with increasing extraction time. After 84 h of extraction, the content of total flavanols measured in the staghorn sumac extract was, on average, 209.01 mg/mL higher compared to the initial value (62.08 mg/mL extract) (Supplementary Table S3, Figure 1C). Procyanidin derivatives accounted for more than 70% of the total flavanol content in the aqueous staghorn sumac extract. Komes et al. [40] also found that the success of the phenolic compound extraction (especially flavanols) from plant tissue in an aqueous solvent depends mainly on extraction duration. In the extract of Himalayan balsam, the flavanols reached their maximum content slightly earlier, after 48 h of extraction (T4, 97.55 mg/mL). However, their content decreased by 50% after 84 h of extraction compared to T4 (Figure 2C, Supplementary Table S4). Moreover, Ko et al. [41] also reported that flavanol derivatives in green tea leaves significantly decreased with extended extraction time.

The content of total flavanones in the aqueous Himalayan balsam extract differed significantly between different extraction times (Figure 2D, Supplementary Table S4). At the beginning of the experiment, the average minimum content of total flavanones (4.53 mg/mL of extract) was 4.9 times lower than the maximum content measured after 48 h of extraction (22.32 mg/mL).

The analysis of the influence of extraction time on the efficiency of flavonol glycoside extraction in staghorn sumac and Himalayan balsam (see Supplementary Tables S3 and S2) shows that the total flavonol content increases significantly with longer extraction times. In the extract of staghorn sumac, the highest extraction efficiency of flavonol derivatives was observed at 12 h, with a 2.4-fold increase in flavonol content compared to the lowest average value at the beginning of the experiment (296 mg/mL) (see Figure 1D, Supplementary Table S3). In contrast, the extraction time for flavonols from Himalayan balsam was significantly longer. Thus, the best extraction of flavonols was achieved after 48 and 84 h when the content was 3 to 3.4 times higher compared to the first time point (1 h) (Figure 2E, Supplementary Table S4). The observed response in flavonol extraction from Himalayan balsam is primarily due to the extraction of quercetin-3-rutinoside and quercetin-3-galactoside, which are the predominant flavonols in this plant and account for about 80% of the total flavonol content. In contrast, flavonol extraction from staghorn sumac is largely influenced by the presence of myricetin-3-rhamnoside and various quercetin derivatives, which together account for a significant proportion of the flavonols in staghorn sumac tissue (approximately 80% of total flavonols) (Supplementary Table S3). The extraction efficiency of flavonol glycosides primarily depends on the number of hydroxyl groups attached to the molecule [42]. Flavonols with fewer OH groups in their structure can be extracted better. Moreover, the extraction efficiency of flavonols increases with extended extraction time [42].

Extraction time also had a considerable influence on the content of the total analyzed phenolics (TAP) in the aqueous extracts of staghorn sumac and Himalayan balsam. With the longest extraction time (84 h), the TAP content in the staghorn sumac extract increased 3.5-fold compared to the lowest content measured at the beginning of the experiment (1 h, 446 mg/mL extract) (Figure 1E, Supplementary Table S3). In the extract of Himalayan balsam, the highest concentration of total phenolics was reached within 48 h after extraction, which represents a fourfold increase compared to the first measurement after 1 h (Figure 2F, Supplementary Table S4). After 84 h of extraction, the total phenolic content decreased to 40% of the previous content.

Earlier scientific research by Kossah et al. [43] found that the optimal extraction time for staghorn sumac fruit is five hours, as this time period maximizes the yield of total polyphenolics. However, extending the extraction time beyond five hours leads to a decrease in the total polyphenolic content. Conversely, Kuźma et al. [44] found that extraction times of 30 or 60 min did not significantly impact the total polyphenolic content in the extraction of parsley leaves.

Based on this study’s findings and prior research [43,44], it can be concluded that extraction time has a varied impact on the extraction efficiency of individual phenolic compounds. Generally, the effectiveness of extracting phenolics from plant tissue improves with longer extraction times. However, the optimum extraction time can vary greatly depending on the phenolic compounds present. In certain cases, a longer extraction time can lead to a decrease in the phenolic content of the plant extract. This reduction is likely due to the different stability of individual phenolic compounds over time. To illustrate, the total polyphenolic content in the extract of Himalayan balsam decreased with extended extraction time, which, according to Kuźma et al. [44], could be due to enzymatic degradation, oxidation of polyphenolic compounds, and the polymerization of insoluble compounds.

## 3. Materials and Methods

### 3.1. Plant Material

Staghorn sumac shoots with leaves were collected on October 8 (between 4 and 5 pm), 2020 in Ljubljana at a location with geographical coordinates 46° 2′ 54.00′′ N, 14° 28′ 31.35′′ E, Himalayan balsam was collected nearby, at a location with geographical coordinates 46° 3′ 7.61′′ N, 14° 28′ 15.29′′ E. The plants were collected randomly within a radius of 300 m around the given coordinate. Staghorn sumac shoots were collected with leaves, whereas Himalayan balsam shoots were collected with leaves, flowers, and fruits. The Himalayan balsam plants were in the phenophase of fruit ripening, and the staghorn sumac plants were in the fruit development phase. The plant material was collected in black PVC bags in four replicates, with one replicate consisting of about 20 plants in the case of Himalayan balsam or 20 shoots in the case of staghorn sumac. After collection, the samples were immediately transported to the laboratory.

### 3.2. Preparation of Aqueous Extracts

To prepare water extracts, all collected plant parts were finely chopped and mixed with water. The mixture was ground well with a grinder. Two concentrations were prepared for each plant species. For vinegar, concentration 1 contained 0.16 g of material per milliliter of water (40 g of plant material + 250 mL of water), whereas concentration 2 contained 0.08 g/mL (20 g of material plus 250 mL of water). For the intact gland, concentration 1 contained 0.25 g/mL (50 g plant material + 200 mL water), whereas concentration 2 contained 0.125 g/mL (25 g material + 200 mL water). The plant suspensions were shaken on a shaker for 60 min at room temperature. After this time, the suspensions were transferred to a refrigerator set at 4 °C. After 11 h, the aqueous extracts were filtered through cotton gauze and then used to treat the seeds; afterward, the germination test was carried out.

### 3.3. Seed Germination

The efficacy of seed germination inhibition was tested on perennial ryegrass (*Lolium perenne* L.). Twenty seeds of perennial ryegrass were germinated on filter paper in Petri dishes. The experiments were carried out in four replicates (N = 80). The seeds in the Petri dishes were watered with 5 mL of the aqueous extract. The Petri dishes were then left at room temperature in a bright room, and after two days, the seeds were watered with another 4 mL of the extract. In the control treatment, the seeds were watered with tap water. After five days, the germinated seeds were counted, and the length of the shoots and roots was measured. A seed was considered germinated if it had a shoot or root with a length of at least 1 mm.

### 3.4. Effect of Extraction Time on Phenolic Compounds from Plant Material

The water extracts from the invasive species prepared to examine the inhibition of seed germination were also used in a study that investigated how phenolic content changes over time. This experiment used the aqueous extracts of staghorn sumac and Himalayan balsam with concentration 2. For each invasive species, four replicates were prepared. The suspensions were shaken on a shaker for 1 h (TERM 1 (T1) = extraction 1 h) and then stored in closed bottles in a refrigerator at 4 °C for different periods of time: 11 h (T2 = 1 h + 11 h), 23 h (T3 = 1 h + 23 h), 47 h (T4 = 1 h + 47 h), and 83 h (T5 = 1 h + 83 h). The extracts were then filtered through gauze, centrifuged, and filtered again through cellulose filters into vials. The vials were stored in a freezer at −20 °C until analysis.

### 3.5. Extraction Efficiency of Phenolic Compound from Plant Material in Different Solvents

Two different solvents were used for the extraction of phenolic compounds from staghorn sumac and Himalayan balsam. To compare the efficiency of phenolic substance extraction from both invasive plant species, two types of solvents were used: water, as the cheapest, most accessible, and non-hazardous solvent, and 80% methanol (80/20 methanol/water), which the literature widely reports as one of the most effective solvents for extracting phenolic substances from plant tissues [45,46]. For methanolic extract, two grams of plant material were mixed with 8 mL of 80% methanol. All sample weights and amounts of solvent used were accurately recorded. For each invasive species, four replicates were prepared. The test tubes were thoroughly shaken and then placed in an ice-cold ultrasonic water bath for 60 min. Afterward, the test tubes were stored in a refrigerator at 4 °C for 11 h. After a total of 12 h of extraction, the alcoholic extracts were centrifuged (Eppendorf Centrifuge 5810 R; Hamburg, Germany), and the supernatant was filtered through PTFE filters (Macherey-Nagel, Düren, Germany) and transferred into vials, which were kept in a freezer at −20 °C until analysis.

The water extracts were prepared earlier, during the germination test, using an extract with concentration 2. For each invasive plant, four replicates were made. After 12 h of extracting the plant material, a few milliliters of the suspension were filtered through gauze and then through cellulose filters into vials. These vials were then stored in a freezer until HPLC analysis.

### 3.6. HPLC-DAD Analysis and Identification of Phenolic Compounds

Phenolic compounds were analyzed employing HPLC (Dionex UltiMate 3000, Thermo Scientific, Waltham, MA, USA) equipped with a DAD detector and a Gemini C18 column (Phenomenex) heated to 25 °C. The compounds were detected at the wavelengths of 280, 350, and 530 nm. Two mobile phases were used to separate the phenolic compounds: A (0.1% formic acid/3% acetonitrile/96.9% double distilled water) and B (0.1% formic acid/3% double distilled water/96.9% acetonitrile), which were mixed according to the gradient program described by Mikulic-Petkovsek et al. [47]. The linear elution gradient was as follows: 5–20% B from 0 to 15 min, then 20–30% B from 15 to 25 min, followed by a transition to 30–90% B from 25 to 30 min, 90–100% B from 30 to 35 min, and 100% B from 35 to 40 min. It was then returned to the initial conditions within 5 min and the column was re-equilibrated for the following 10 min. The samples were injected in a volume of 20 μL, and the flow rate of the mobile phase was set to 0.6 mL/min. The individual metabolites were identified by mass spectrometry (LTQ XL Linear Ion Trap Mass Spectrometer, Thermo Scientific, Waltham, MA, USA) with electrospray ionization (ESI) in negative and positive modes, as described by Mikulic-Petkovsek et al. [47]. The phenolic compounds were confirmed based on the fragmentation products, the comparison of retention times with the corresponding standards, and the comparison of the spectrum of the individual peaks with the standard samples. The contents of phenolic compounds were calculated using the peak areas of the samples and the corresponding standard curves for phenolic substances. The contents of phenolic compounds in the invasive species studied were expressed in μg/g or mg/g dry weight (DW).

### 3.7. Reagents

For the quantification and identification of hydroxycinnamic acid derivatives in both invasive species, the following chemicals were used: *p*-coumaric acid and ferulic acid from Fluka Chemie and caffeic and 3-caffeoylquinic acid from Sigma-Aldrich. For hydroxybenzoic acids, gallic acid, protocatechuic acid, and vanillic acid from Sigma Aldrich (Steinheim, Germany) were used. For flavanols, procyanidin B1, catechin, and epicatechin from Fluka Chemie (Buchs, Switzerland) were used. For flavonols, the following acids were used: quercetin-3-galactoside, quercetin-3-glucoside, and quercetin-3-rhamnoside from Sigma Aldrich; kaempferol-3-glucoside and quercetin-3-rutinoside from Fluka Chemie; quercetin-3-xyloside, quercetin-3-arabinopyranoside, myricetin-3-rutinoside, and quercetin-3-arabinofuranoside from Apin Chemicals LTD (Abingdon, UK); isorhamnetin-3-glucoside from Extrasynthèse (Genay Cedex, France). For flavanones, naringenin from Fluka Chemie was used. For anthocyanins, cyanidin-3-glucoside from Fluka, malvidin-3-glucoside from Sigma Aldrich, and delphinidin-3-glucoside from Extrasynthèse were used. To estimate the content of phenolic compounds for which no reference standards were available, their concentrations were determined using a chemically similar phenolic compound. For the preparation of the extraction solution for phenolic compounds, HPLC-MS grade methanol and formic acid from Sigma-Aldrich were used. For both mobile phases, the following were mixed: acetonitrile-HPLC-MS from JT Baker, formic acid from Sigma-Aldrich, and double-distilled water purified with a Milli-Q system (Millipore, Bedford, MA, USA).

### 3.8. Statistical Analysis

The R Commander program R 3.6.1 was used for the statistical analysis of the data. A one-way analysis of variance (ANOVA) was used to analyze the germination test and the effect of extraction time on the phenolic compound content. Statistical differences between the two solvents (methanol and water) were determined using the *t*-test (95% confidence interval). For ANOVA of the characteristic features, multiple comparisons were performed using Tukey’s HSD test. All mean values of the results, together with the standard error, are shown in the tables and figures, as well as the differences between the treatments, which are indicated by different letters.

## 4. Conclusions

Invasive plant species (IPS) are acknowledged as the second-greatest worldwide threat to biodiversity. However, they can also serve as a cost-effective source of bioactive substances, which can be utilized for various pharmacological purposes or as biopesticides. Invasive species hold great potential as a source of phytochemicals that could reduce the growth of some economically important weeds. The first step in utilizing these plants is to obtain a phytochemically enriched extract from IPS for further use. To optimize the yield of bioactive compounds, different solvents can be employed during the extraction process to ensure maximum yield of the desired phytochemicals. Water is the first choice of solvent, as it is environmentally friendly and inexpensive. Although water is generally less effective than alcohol, high concentrations of phenolic compounds can still be successfully extracted, especially when the extraction time is extended to 48 or 84 h. Notably, water extraction was more effective for extracting hydroxybenzoic acids, hydroxycinnamic acids, and flavonols from staghorn sumac. The results of this study indicate that aqueous extracts from staghorn sumac and Himalayan balsam exhibited inhibitory effects on the seed germination of perennial ryegrass and substantially suppressed the growth of its shoots.

The diversity of secondary metabolites present in the extracts of both staghorn sumac and Himalayan balsam, each with different modes of action, indicates their potential as bioherbicides. These bioactive compounds from IPS could offer valuable applications in agriculture and horticulture in the future. Consequently, the populations of invasive species in the environment would decline, which would positively impact the growth of native species and their biodiversity. Future studies should focus on evaluating the allelopathic effects on a broader range of plant species, particularly on weed species that pose a significant economic threat to agricultural productivity.

## Figures and Tables

**Table 1:** The content of individual phenolic compounds and total phenolic groups (mean ± standard error in μg/g DW) in the staghorn sumac extract.

Phenolic Compound	Alcoholic Extract	Water Extract
Digalloyl hexoside	14.94 ± 1.83 a	6.49 ± 2.69 b
Gallic acid	1007.49 ± 88.53 b	5532.30 ± 411.82 a
Gallic acid hexose derivative	54.85 ± 2.53	89.64 ± 18.01
Galloyl hexose 1	22.51 ± 3.92	19.12 ± 9.81
Galloyl hexose 2	440.13 ± 53.20	382.46 ± 39.80
Galloylquinic acid 1	76.18 ± 3.50	124.50 ± 25.01
Methyl gallate	344.25 ± 9.77 b	531.59 ± 57.03 a
Protocatechuic acid hexoside	166.01 ± 20.32 a	72.11 ± 29.90 b
Syringic acid hexoside	243.52 ± 26.27	320.53 ± 49.08
**Total hydroxybenzoic acid derivatives**	2369.88 ± 170.11 b	7078.75 ± 565.29 a
3-*p*-coumaroylquinic acid	48.86 ± 2.81	58.68 ± 5.16
3-feruloylquinic acid	74.85 ± 1.34 b	51.40 ± 5.69 a
3-caffeoylquinic acid	150.06 ± 6.86 b	376.47 ± 15.32 a
4-*p*-coumaroylquinic acid	29.52 ± 4.56	14.32 ± 5.09
5-*p*-coumaroylquinic acid 1	11.08 ± 2.03	13.15 ± 4.36
5-*p*-coumaroylquinic acid 2	21.12 ± 4.05	11.57 ± 2.86
5-caffeoylquinic acid	8.70 ± 1.05	7.56 ± 0.79
Caffeic acid	50.37 ± 7.64	29.86 ± 8.08
Caffeic acid hexoside 1	14.91 ± 0.68 b	37.42 ± 1.52 a
Caffeic acid hexoside 2	38.10 ± 2.92 a	19.85 ± 2.18 b
*p*-coumaric acid hexoside 1	35.46 ± 1.62 b	88.97 ± 3.62 a
*p*-coumaric acid hexoside 2	20.12 ± 1.54 a	10.48 ± 1.15 b
**Total hydroxycinnamic acid derivatives**	503.14 ± 21.25 b	719.72 ± 44.61 a
Catechin	965.42 ± 55.62	1159.52 ± 101.91
Procyanidin derivative	1215.37 ± 152.92 a	1162.43 ± 266.49 a
Procyanidin dimer 1	295.69 ± 51.47	322.74 ± 102.84
Procyanidin dimer 2	1691.20 ± 129.65 a	880.81 ± 96.59 b
Procyanidin dimer 3	173.75 ± 26.85	84.26 ± 29.96
Procyanidin dimer 4	621.31 ± 119.16	340.50 ± 84.22
Procyanidin trimer 1	233.35 ± 13.44	280.26 ± 24.63
Procyanidin trimer 2	1450.09 ± 200.49	856.08 ± 176.39
Procyanidin trimer 3	260.79 ± 47.71	309.62 ± 102.57
**Total flavanols**	6906.97 ± 522.96	5396.22 ± 954.83
Isorhamnetin hexoside	91.16 ± 4.66	94.19 ± 2.25
Kaempferol hexoside	297.83 ± 6.48	283.21 ± 7.50
Kaempferol hydroxyhexoside	275.39 ± 17.97	345.00 ± 28.83
Kaempferol pentoside 1	158.39 ± 4.17 a	139.67 ± 2.21 b
Kaempferol pentoside 2	160.19 ± 4.13 a	120.08 ± 5.41 b
Laricitrin hexoside	361.42 ± 8.93 a	318.37 ± 5.94 b
Myricetin hexoside 1	473.23 ± 16.23 b	700.24 ± 8.39 a
Myricetin hexoside 2	706.75 ± 17.59 b	985.94 ± 9.31 a
Myricetin pentoside 1	208.40 ± 6.46 a	218.30 ± 3.90 a
Myricetin pentoside 2	119.39 ± 3.07 a	107.16 ± 3.08 b
Myricetin ramnoside	6520.14 ± 181.92 b	9817.58 ± 92.46 a
Quercetin-3-arabinofuranoside	729.92 ± 17.60 b	1049.67 ± 27.07 a
Quercetin-3-arabinopyranoside	303.45 ± 6.60	288.55 ± 7.65
Quercetin-3-galactoside	1163.80 ± 173.60	1465.73 ± 14.99
Quercetin-3-glucoside	1412.93 ± 157.19 b	2458.04 ± 78.27 a
Quercetin-3-ramnoside	3587.43 ± 44.54 b	3994.97 ± 105.66 a
Quercetin-3-rutinoside	3.52 ± 0.09 a	3.16 ± 0.09 b
Quercetin-3-xyloside	268.73 ± 4.21 a	228.98 ± 3.84 b
**Total flavonols**	16,842.03 ± 341.31 b	22,628.20 ± 264.48 a

Different letters in the row indicate statistical differences in the average content of the individual phenolic compounds and phenolic groups between the solvents, calculated with the *t*-test (*p* ≤ 0.05).

**Table 2:** The content of individual phenolic compounds and total phenolic groups (mean ± standard error in μg/g DW) in the Himalayan balsam extract.

Phenolic Group	Alcoholic Extract	Water Extract
Cyanidin-coumaroylhexoside	114.67 ± 10.58	/
Cyanidin-malonylhexoside	27.52 ± 2.54	/
Delphinidin-coumaroylhexoside	86.16 ± 7.49	/
Delphinidin-malonylhexoside	53.42 ± 4.65	/
Malvidin-malonylhexoside	13.58 ± 3.51	/
Malvidin-coumaroylhexoside	15.61 ± 4.04	/
**Total anthocyanins**	310.96 ± 27.86	0.00 ± 0.00
Protocatechuic acid	1056.92 ± 10.82 a	252.21 ± 18.45 b
Vanillic acid	150.99 ± 1.55 a	36.03 ± 2.64 b
**Total hydroxybenzoic acid derivatives**	1207.90 ± 12.37 a	288.24 ± 21.08 b
Ferulic acid 1	121 ± 7.74 a	43.3 ± 3.12 b
Ferulic acid 2	51.1 ± 1.90 a	2.59 ± 0.49 b
*p*-Coumaric acid hexoside	122 ± 2.73 a	11.8 ± 2.63 b
Caffeic acid 1	535 ± 51.0 a	134 ± 9.06 b
Caffeic acid 2	337 ± 21.4 a	119 ± 8.63 b
*p*-Coumaric acid 1	145 ± 9.22 a	51.6 ± 3.72 b
*p*-Coumaric acid 2	86.0 ± 1.94 a	11.6 ± 4.20 b
**Total hydroxycinnamic acid derivatives**	1399.08 ± 90.70 a	374.91 ± 29.95 b
Epicatechin	1854.47 ± 117.76 a	658.77 ± 47.48 b
Catechin	667.68 ± 63.59 a	167.43 ± 11.30 b
Procyanidin dimer 1	524.02 ± 11.69 a	50.65 ± 11.24 b
Procyanidin dimer 2	1007.95 ± 109.74 a	406.99 ± 82.98 b
**Total flavanols**	4054.12 ± 271.01 a	1283.83 ± 123.44 b
Eriodictyol hexoside 1	3685.05 ± 136.97 a	187.06 ± 35.33 b
Eriodictyol hexoside 2	194.44 ± 14.17	186.74 ± 40.39
Naringenin hexoside	73.45 ± 3.12 a	36.51 ± 5.16 b
**Total flavanones**	3952.94 ± 151.20 a	410.31 ± 39.32 b
Isorhamnetin-3-rutinoside	3685.05 ± 136.97 a	187.06 ± 35.33 b
Kaempferol-3-rutinoside	194.44 ± 14.17	186.74 ± 40.39
Kaempferol acetyl hexoside	73.45 ± 3.12 a	36.51 ± 5.16 b
Kaempferol hexoside 1	87.50 ± 16.89	55.35 ± 11.62
Kaempferol hexoside 2	1772.39 ± 279.59 a	23.14 ± 5.42 b
Kaempferol rhamnosyl dihexoside	44.58 ± 2.50 a	2.86 ± 0.76 b
Quercetin-3-galactoside	607.83 ± 34.10 a	39.04 ± 10.37 b
Quercetin malonyl hexoside	975.49 ± 167.96 a	42.38 ± 10.36 b
Quercetin-3-glucoside	87.50 ± 16.89	55.35 ± 11.62
Quercetin-3- rutinoside	1772.39 ± 279.59 a	23.14 ± 5.42 b
Myricetin-3-glucuronide	18.92 ± 2.45	/
**Total flavonols**	6040.57 ± 760.72 a	695.88 ± 109.12 b

Different letters in the row indicate statistical differences in the average content of the individual phenolic compounds and phenolic groups between the solvents, calculated with the *t*-test (*p* ≤ 0.05).

**Table 3:** The seed germination of perennial ryegrass (%) and shoot and root length (mm) (mean ± standard error) after treatment with the staghorn sumac and Himalayan balsam extracts.

	Concentration (g/mL)	Label	Germination (%)	Shoot Length (mm)	Root Length (mm)
Control			97.50 ± 1.71 a	28.03 ± 1.09 a	20.57 ± 0.88 b
Staghorn sumac	0.16	1	18.75 ± 5.54 d	11.20 ± 0.96 c	1.33 ± 0.16 c
	0.08	2	17.50 ± 4.33 d	12.86 ± 1.54 c	2.07 ± 0.50 c
Himalayan balsam	0.25	3	42.50 ± 4.79 c	13.38 ± 1.50 c	19.18 ± 1.61 b
	0.125	4	71.25 ± 8.75 b	20.29 ± 1.41 b	26.26 ± 1.30 a

Different letters in the column indicate statistically significant differences in the average germination, shoot length, and root length of perennial ryegrass between plant water extracts and control (water), determined with Tukey’s HSD test (*p* ≤ 0.05).

## Data Availability

The data presented in this study are available in the article and the Supplementary Material.

## Supplementary Materials

The following supporting information can be downloaded at https://www.mdpi.com/article/10.3390/plants13233339/s1: Table S1. Identification of phenolic compounds in staghorn sumac in negative mode with HPLC-MS and MS^2^/MS^3^. Table S2. Identification of phenolic compounds in Himalayan balsam in negative and positive mode with HPLC-MS and MS^2^/MS^3^. Table S3. The content of phenolic compounds and standard error (mg/mL) in the staghorn sumac water extract in different extraction times. Different letters in the row indicate statistical differences in the content of individual phenolic compounds or phenolic groups between different extraction times (Tukey HSD test). (T1 = 1 h of the extraction, T2 = 12 h of the extraction, T3 = 24 h of the extraction, T4 = 48 h of the extraction, and T5 = 84 h of the extraction). Table S4. The content of phenolic compounds and standard error (mg/mL) in the Himalayan balsam water extract in different extraction times. Different letters in the row indicate statistical differences in the content of individual phenolic compounds or phenolic groups between different extraction times (Tukey HSD test). (T1 = 1 h of the extraction, T2 = 12 h of the extraction, T3 = 24 h of the extraction, T4 = 48 h of the extraction, and T5 = 84 h of the extraction). Figure S1. Chromatograms of staghorn sumac extract recorded at A 280 nm and B 350 nm. Peak numbers are described in Table S1. Figure S2. Chromatograms of Himalayan balsam extract recorded at A 530 nm, B 280 nm, and C 350 nm. Peak numbers are described in Table S2.
