# Supplementary material for: Identification of Phenolic Compounds in the Invasive Plants Staghorn Sumac and Himalayan Balsam: Impact of Time and Solvent on the Extraction of Phenolics and Extract Evaluation on Germination Inhibition
Source: Plants (Basel). 2024 Nov 28;13(23):3339. doi: 10.3390/plants13233339 (PMC11644323; doi:10.3390/plants13233339)
Supplement: Supplementary file 1 [file plants-13-03339-s001.zip › Supplementary Table S1.pdf]

Supplementary Table S1. Identification of phenolic compounds in staghorn sumac in negative mode with HPLC-MS and MS^2/MS^3

Peak No.	Phenolic compound	**λ (nm)**	**$[M-H]^-$ (*m/z*)**	**MS^2 (*m/z*)**	**MS^3 (*m/z*)**
1	Gallic acid	271	169	125	
2	Gallic acid hexose derivative	276	487	331,169	
3	Galloylquinic acid 1	272	343	169,191	
4	Syringic acid hexoside	263	359	197	
5	Protocatechuic acid hexoside	259,263	315	153,109	
5	Digalloyl hexoside	270	483	331,169	
6	Caffeic acid hexoside 1	330	341	179	
7	3-caffeoylquinic acid	234,326	353	191,179,135	173,127,85
7	*p*-coumaric acid hexoside 1	322,275	325	163,119	
8	Galloyl hexose 1	276	331	169	
9	Procyanidin dimer 1	278	577	425,451,407,289	
10	Caffeic acid hexoside 2	330	341	179	
10	*p*-coumaric acid hexoside 2	322,275	325	163,119	
10	Procyanidin dimer 2	278	577	425,451,407,289	
11	Methyl gallate	277	183	168,124	
12	3-*p*-coumaroylquinic acid	312	337	163	
12	Catechin	234,279	289	245	
12	Procyanidin trimer 1	235,279	865	577,425,407,289	
13	5-caffeoylquinic acid	234,328	353	191,179,135	173,127,85
14	3-feruloylquinic acid	320	367	193,134	149
15	Galloyl hexose 2	276	331	169	
16	Caffeic acid	323	179	135	
17	4-*p*-coumaroylquinic acid	312	337	173,163,155,137,191	
17	Procyanidin trimer 2	234,279	865	577,425,407,289	
18	Procyanidin dimer 3	279	577	425,407,289	
19	5-*p*-coumaroylquinic acid 1	312	337	191,173,163	
20	Procyanidin trimer 3	235,280	865	577,425,289	
21	Procyanidin derivative	234,279	729	577,559,593,451,425,407,289	
22	5-*p*-coumaroylquinic acid 2	311	337	191,163,173	
22	Procyanidin dimer 4	278	577	425,451,289	
23	Myricetin hexoside 1	256,349	479	317	
24	Myricetin hexoside 2	256,350	479	317	
25	Myricetin pentoside 1	266,352	449	317	
26	Myricetin pentoside 2	266,352	449	317	
26	Quercetin-3-rutinoside	255,355	609	301	
27	Myricetin rhamnoside	265,350	463	317	
28	Quercetin-3-galactoside	256,356	463	301	
29	Quercetin3-glucoside	255,355	463	301	
30	Quercetin-3-xyloside	356,255	433	301	
31	Kaempferol hexoside	266,346	447	285	
31	Quercetin-3-arabinopyranoside	256,352	433	301	
32	Quercetin-3-arbinofuranoside	355,255	433	301	
33	Quercetin-3-rhamnoside	266,356	447	301	
34	Kaempferol pentoside 1	265,348	417	285	
35	Laricitrin hexoside	245,286	493	331	
36	Kaempferol pentoside 2	265,349	417	285	
37	Unknown	266,352	697	621,545	
38	Kaempferol hydroxyhexoside	264,347	465	303,285	
39	Isorhamnetin hexoside	255,367	477	315	
