# Supplementary material for: Identification of Phenolic Compounds in the Invasive Plants Staghorn Sumac and Himalayan Balsam: Impact of Time and Solvent on the Extraction of Phenolics and Extract Evaluation on Germination Inhibition
Source: Plants (Basel). 2024 Nov 28;13(23):3339. doi: 10.3390/plants13233339 (PMC11644323; doi:10.3390/plants13233339)
Supplement: Supplementary file 1 [file plants-13-03339-s001.zip › Supplementary Table S2.pdf]

Supplementary Table S2. Identification of phenolic compounds in Himalayan balsam in negative and positive mode with HPLC-MS and MS^2/MS^3

**Peak No.**	**Phenolic group**	**λ (nm)**	**$[M-H]^-$ (*m/z*)**	**MS^2 (*m/z*)**	**MS^3 (*m/z*)**
1	Delphinidin-coumaroylhexoside	279,518	611	449,303	
1	Delphinidin-malonylhexoside	280, 523	551	303	
2	Cyanidin-coumaroylhexoside	278, 520	595	433,287	
2	Cyanidin-malonylhexoside	279, 521	535	287	
3	Malvidin-malonylhexoside	279,516	579	331	
3	Malvidin-coumaroylhexoside	278,522	655	331	
4	Protocatechuic acid	259,264	153	109	
4	Vanillic acid	259,290	167	123,108	
5	*p*-Coumaric acid hexoside	322,275	325	163	
6	Procyanidin dimer 1	234,278	577	425,407,289	
7	Caffeic acid 1	321	179	135	
8	Catechin	234,279	289	245	
9	Procyanidin dimer 2	234,278	577	425,407,289	
10	Caffeic acid 2	321	179	135	
11	Epicatechin	234,279	289	245	
12	Ferulic acid 1	322	193	134,149,178	
13	*p*-Coumaric acid 1	310	163	119	
14	Ferulic acid 2	322	193	134,149,178	
14	Eriodictyol hexoside 1	283,328	449	287	
15	*p*-Coumaric acid 2	310	163	119	
16	Eriodictyol hexoside 2	283,328	449	287	
17	Naringenin hexoside	225, 289	433	271	
18	Quercetin-3-rutinoside	255,355	609	301	
19	Kaempferol rhamnosyl dihexoside	263, 343	755	609	285
20	Quercetin-3-galactoside	256,356	463	301	
21	Quercetin-3-glucoside	255,355	463	301	
21	Myricetin-3-glucuronide	266,352	493	317	
22	Isorhamnetin-3-rutinoside	255,351	623	315	
22	Kaempferol-3-rutinoside	264,345	593	285	
23	Kaempferol hexoside 1	266,346	447	285	
24	Kaempferol hexoside 2	266,346	447	285	
25	Quercetin malonyl hexoside	255,355	549	505,301	
26	Kaempferol acetyl hexoside	265,344	489	285	

*$[M]^+$ (*m/z*) anthocyanins were obtained in the positive ion mode, other phenolics in the negative ion mode
