# Supplementary material for: Identification of Phenolic Compounds in the Invasive Plants Staghorn Sumac and Himalayan Balsam: Impact of Time and Solvent on the Extraction of Phenolics and Extract Evaluation on Germination Inhibition
Source: Plants (Basel). 2024 Nov 28;13(23):3339. doi: 10.3390/plants13233339 (PMC11644323; doi:10.3390/plants13233339)
Supplement: Supplementary file 1 [file plants-13-03339-s001.zip › Supplementary Table S3.pdf]

Supplementary Table S3.The content of phenolic compounds and standard error (mg/ml) in the staghorn sumac water extract in different extraction times. Different letters in the row indicate statistical differences in the content of individual phenolic compounds or phenolic groups between different extraction times (Tukey HSD test). (T1 = 1 hour of the extraction, T2 = 12 hours of the extraction, T3 = 24 hours of the extraction, T4 = 48 hours of the extraction and T5 = 84 hours of the extraction).

Phenolic group	T1	T2	T3	T4	T5
**Hydroxybenzoic acids derivatives**	**75.64 ± 3.98 c**	**226.56 ± 18.04 b**	**253.59 ± 21.62 b**	**310.75 ± 9.50 b**	**538.30 ± 31.29 a**
Gallic acid hexose derivate	1.05 ± 0.22 b	2.87 ± 0.58 b	1.89 ± 0.22 b	2.95 ± 0.50 b	5.54 ± 0.62 a
Digalloylhexoside	0.08 ± 0.01 b	0.21 ± 0.09 ab	0.14 ± 0.02 b	0.18 ± 0.08 ab	0.46 ± 0.11 a
Gallic acid	54.74 ± 3.41 d	176.93 ± 13.17 c	214.76 ± 24.13 bc	264.61 ± 6.03 b	460.64 ± 28.29 a
Galloylhexose 1	0.07 ± 0.05 b	0.79 ± 0.25 b	0.55 ± 0.21 b	0.88 ± 0.35 b	2.23 ± 0.48 b
Galloylhexose 2	9.04 ± 0.95 b	12.23 ± 1.27 b	10.75 ± 0.11 b	11.68 ± 1.76 b	19.58 ± 2.53 a
Galloylquinic acid 1	1.46 ± 0.31 b	3.98 ± 0.80 b	2.62 ± 0.31 b	4.09 ± 0.70 b	7.70 ± 0.85 a
Protocatechuic acid hexoside	0.94 ± 0.09 b	2.31 ± 0.96 ab	1.50 ± 0.23 b	2.01 ± 0.90 ab	5.15 ± 1.17 a
Syringic acid hexoside	3.74 ± 0.40 c	10.25 ± 1.57 ab	6.91 ± 1.08 bc	8.54 ± 1.53 abc	14.21 ± 1.76 a
Methyl gallate	4.52 ± 0.37 c	17.00 ± 1.82 ab	14.49 ± 1.08 b	15.81 ± 1.37 b	22.78 ± 1.72 a
**Hydroxycinnamic acid derivatives**	**12.33 ± 0.74 b**	**34.00 ± 4.24 a**	**31.63 ± 4.50 ab**	**36.22 ± 5.88 a**	**51.68 ± 6.40 a**
3-*p*-coumaroylquinic acid	0.92 ± 0.13 c	1.88 ± 0.17 ab	1.37 ± 0.09 bc	1.35 ± 0.09 bc	2.34 ± 0.20 a
3-caffeoylquinic acid	4.87 ± 0.43 b	12.04 ± 0.49 a	10.07 ± 1.05 a	11.33 ± 0.89 a	12.44 ± 1.42 a
3-feruloylquinic acid	0.81 ± 0.17 c	1.64 ± 0.18 bc	1.63 ± 0.21 bc	2.46 ± 0.43 b	3.77 ± 0.39 a
4-*p*-coumaroylquinic acid	0.35 ± 0.11 a	0.46 ± 0.16 a	0.42 ± 0.10 a	0.47 ± 0.10 a	0.76 ± 0.13 a
5-*p*-coumaroylquinic acid 1	0.06 ± 0.02 b	0.42 ± 0.14 ab	0.36 ± 0.08 ab	0.39 ± 0.07 ab	0.75 ± 0.09 a
5-*p*-coumaroylquinic acid 2	0.25 ± 0.02 c	0.37 ± 0.09 bc	0.56 ± 0.04 ab	0.47 ± 0.06 abc	0.65 ± 0.07 a
5-caffeoylquinic acid	0.18 ± 0.02 b	0.24 ± 0.03 b	0.21 ± 0.00 b	0.23 ± 0.04 b	0.39 ± 0.05 a
Caffeic acid	2.98 ± 0.30 b	11.94 ± 3.23 ab	12.94 ± 2.87 ab	14.93 ± 4.01 ab	25.30 ± 4.06 a
Caffeic acid hexoside 1	0.48 ± 0.04 b	1.20 ± 0.05 a	1.00 ± 0.11 a	1.13 ± 0.09 a	1.24 ± 0.14 a
Caffeic acid hexoside 2	0.19 ± 0.02 c	0.63 ± 0.07 ab	0.45 ± 0.05 b	0.51 ± 0.06 ab	0.72 ± 0.05 a
p-coumaric acid hexoside 1	1.15 ± 0.10 b	2.85 ± 0.12 a	2.38 ± 0.25 a	2.68 ± 0.21 a	2.94 ± 0.34 a
p-coumaric acid hexoside 2	0.10 ± 0.01 c	0.34 ± 0.04 ab	0.24 ± 0.03 b	0.27 ± 0.03 ab	0.38 ± 0.03 a

It continues

Phenolic group	T1	T2	T3	T4	T5
**Flavanols**	**62.08 ± 5.04 c**	**172.58 ± 30.54 b**	**147.06 ± 17.17 bc**	**160.95 ± 18.25 b**	**271.09 ± 29.56 a**
Catechin	18.27 ± 2.53 c	37.08 ± 3.26 ab	27.15 ± 1.73 bc	26.61 ± 1.83 bc	46.18 ± 3.96 a
Procyanidin derivate	8.04 ± 2.18 b	37.18 ± 8.52 a	34.05 ± 4.81 a	41.02 ± 4.53 a	56.73 ± 7.15 a
Procyanidin dimer 1	0.96 ± 0.62 b	10.32 ± 3.29 b	7.26 ± 2.71 b	11.63 ± 4.59 b	29.30 ± 6.29 a
Procyanidin dimer 2	8.31 ± 0.84 c	28.17 ± 3.09 ab	19.98 ± 2.39 b	22.79 ± 2.47 ab	32.05 ± 2.13 a
Procyanidin dimer 3	2.03 ± 0.64 a	2.70 ± 0.96 a	2.48 ± 0.59 a	2.74 ± 0.57 a	4.50 ± 0.78 a
Procyanidin dimer 4	7.42 ± 0.57 c	10.89 ± 2.69 bc	16.45 ± 1.29 ab	13.90 ± 1.83 abc	19.24 ± 1.98 a
Procyanidin trimer 1	4.42 ± 0.61 c	8.96 ± 0.79 ab	6.56 ± 0.42 bc	6.43 ± 0.44 bc	11.16 ± 0.96 a
Procyanidin trimer 2	11.19 ± 1.84 b	27.38 ± 5.64 b	24.66 ± 3.45 b	26.61 ± 3.67 b	54.35 ± 6.19 a
Procyanidin trimer 3	1.46 ± 0.56 b	9.90 ± 3.28 ab	8.49 ± 1.84 ab	9.23 ± 1.66 ab	17.60 ± 2.21 a
**Flavonols**	**296.17 ± 26.48 b**	**723.38 ± 8.46 a**	**671.67 ± 37.55 a**	**653.55 ± 25.44 a**	**718.66 ± 13.45 a**
Isorhamnetin hexoside	1.46 ± 0.38 b	3.01 ± 0.07 a	2.33 ± 0.25 ab	2.36 ± 0.30 ab	2.92 ± 0.08 a
Kaempferol hexoside	4.07 ± 0.33 c	9.06 ± 0.24 a	8.11 ± 0.39 ab	7.11 ± 0.23 b	8.41 ± 0.24 a
Kaempferol hydroxy hexoside	7.37 ± 0.82 b	11.03 ± 0.92 ab	8.88 ± 1.29 ab	9.87 ± 0.93 ab	12.91 ± 1.03 a
Kaempferol pentoside 1	2.08 ± 0.30 b	4.47 ± 0.07 a	4.08 ± 0.25 a	3.84 ± 0.14 a	4.41 ± 0.08a
Kaempferol pentoside 2	1.44 ± 0.22 b	3.84 ± 0.17 a	3.44 ± 0.23 a	3.19 ± 0.15 a	3.55 ± 0.09 a
Laricitrin hexoside	5.25 ± 0.61 c	10.18 ± 0.19 ab	9.26 ± 0.55 b	9.07 ± 0.33 b	11.52 ± 0.29 a
Myricetin hexoside 1	9.40 ± 0.77 b	22.40 ± 0.27 a	20.63 ± 1.59 a	19.86 ± 0.64 a	21.58 ± 0.32 a
Myricetin hexoside 2	14.92 ± 1.68 b	31.53 ± 0.30 a	31.90 ± 1.58 a	29.44 ± 0.92 a	30.89 ± 1.09 a
Myricetin pentoside 1	3.18 ± 0.43 b	6.98 ± 0.13 a	6.52 ± 0.47 a	6.23 ± 0.20 a	6.67 ± 0.14 a
Myricetin pentoside 2	1.78 ± 0.26 b	3.43 ± 0.10 a	3.99 ± 0.66 a	2.96 ± 0.11 ab	3.42 ± 0.08 a
Myricetin rhamnoside	122.10 ± 10.30 b	313.98 ± 2.96 a	295.37 ± 17.78 a	287.08 ± 11.73 a	313.19 ± 6.83 a
Quercetin-3-arabinofuranoside	9.19 ± 1.00 c	33.57 ± 0.87 a	29.80 ± 1.44 ab	26.48 ± 1.35 b	28.74 ± 0.50 b

It continues

Phenolic group	T1	T2	T3	T4	T5
Quercetin-3-arabinopyranoside	4.14 ± 0.33 c	9.23 ± 0.25 a	8.26 ± 0.40 ab	7.24 ± 0.23 b	8.57 ± 0.25 a
Quercetin-3-galactoside	21.00 ± 1.83 b	46.88 ± 0.48 a	51.72 ± 4.10 a	45.36 ± 1.53 a	46.65 ± 0.82 a
Quercetin-3-glucoside	29.37 ± 2.28 b	78.61 ± 2.50 a	70.45 ± 3.76 a	68.12 ± 2.73 a	73.75 ± 1.55 a
Quercetin-3-rhamnoside	55.81 ± 7.23 c	127.76 ± 3.38 b	110.32 ± 6.62 ab	118.82 ± 4.69 ab	133.94 ± 2.79 a
Quercetin-3-rutinoside	0.05 ± 0.01 b	0.10 ± 0.00 a	0.12 ± 0.02 a	0.09 ± 0.00 ab	0.10 ± 0.00 a
Quercetin-3-xyloside	3.55 ± 0.51 b	7.32 ± 0.12 a	6.51 ± 0.59 a	6.43 ± 0.24 a	7.46 ± 0.13 a
**Total polyphenolics**	**446.22 ± 34.49 c**	**1156.51 ± 55.63 b**	**1103.96 ± 35.15 b**	**1161.47 ± 53.01 b**	**1579.73 ± 66.07 a**
