# Supplementary material for: Identification of Phenolic Compounds in the Invasive Plants Staghorn Sumac and Himalayan Balsam: Impact of Time and Solvent on the Extraction of Phenolics and Extract Evaluation on Germination Inhibition
Source: Plants (Basel). 2024 Nov 28;13(23):3339. doi: 10.3390/plants13233339 (PMC11644323; doi:10.3390/plants13233339)
Supplement: Supplementary file 1 [file plants-13-03339-s001.zip › Supplementary Table S4.pdf]

Supplementary Table S4. The content of phenolic compounds and standard error (mg/ml) in the Himalayan balsam water extract in different extraction times. Different letters in the row indicate statistical differences in the content of individual phenolic compounds or phenolic groups between different extraction times (Tukey HSD test). (T1 = 1 hour of the extraction, T2 = 12 hours of the extraction, T3 = 24 hours of the extraction, T4 = 48 hours of the extraction and T5 = 84 hours of the extraction).

Phenolic group	T1	T2	T3	T4	T5
**Hydroxybenzoic acids derivatives**	**6.24 ± 0.33 c**	**4.30 ± 0.31 c**	**16.55 ± 1.38 b**	**20.92 ± 0.39 a**	**13.48 ± 1.56 b**
Protocatechuic acid	5.46 ± 0.25 c	3.76 ± 0.28 c	14.48 ± 1.21 b	18.31 ± 0.35 a	11.80 ± 1.36 b
Vanillic acid	0.78 ± 0.04 c	0.54 ± 0.04 c	2.07 ± 0.17 b	2.62 ± 0.05 a	1.69 ± 0.20 b
**Hydroxycinnamic acid derivatives**	**5.92 ± 0.32 c**	**5.59 ± 0.45 c**	**26.08 ± 1.50 a**	**29.34 ± 0.92 a**	**13.04 ± 0.58 b**
Ferulic acid 1	0.97 ± 0.03 d	0.65 ± 0.05 d	2.78 ± 0.11 b	3.38 ± 0.15 a	1.79 ± 0.87 c
Ferulic acid 2	0.03 ± 0.006 c	0.04 ± 0.007 c	0.17 ± 0.02 ab	0.19 ± 0.004 a	0.12 ± 0.01 b
p-coumaric acid hexoside	0.22 ± 0.04 c	0.18 ± 0.04 c	1.30 ± 0.24 b	1.98 ± 0.04 a	0.90 ± 0.12 b
Caffeic acid 1	0.77 ± 0.12 b	2.00 ± 0.14 b	9.68 ± 0.71 a	9.07 ± 0.48 a	2.18 ± 0.23 b
Caffeic acid 2	2.67 ± 0.07 d	1.78 ± 0.13 d	7.70 ± 0.31 b	9.35 ± 0.41 a	4.96 ± 0.24 c
p-coumaric acid 1	1.15 ± 0.03 d	0.77 ± 0.06 d	3.32 ± 0.13 b	4.03 ± 0.18 a	2.14 ± 0.10 c
p-coumaric acid 2	0.10 ± 0.04 b	0.17 ± 0.06 b	1.14 ± 0.19 a	1.33 ± 0.05 a	0.96 ± 0.09 a
**Flavanols**	**22.56 ± 2.84 d**	**19.13 ± 3.68 d**	**83.00 ± 10.57 b**	**97.55 ± 5.27 a**	**49.67 ± 5.01c**
Epicatechin	14.72 ± 0.38 d	9.82 ± 0.71 d	4.24 ± 1.68 b	51.46 ± 2.26 a	27.29 ± 1.32 c
Catechin	0.96 ± 0.16 b	2.50 ± 0.17 b	12.07 ± 0.89 a	11.31 ± 0.59 a	2.71 ± 0.28 b
Procyanidin dimer 1	0.96 ± 0.18 c	0.76 ± 0.17 c	5.56 ± 1.01 b	8.47 ± 0.18 a	3.86 ± 0.51 b
Procyanidin dimer 2	5.93 ± 0.76 c	6.06 ± 1.24 c	23.03 ± 2.81 a	26.31 ± 0.30 a	15.81 ± 1.90 b
**Flavanones**	**4.53 ± 0.41 c**	**6.11 ± 0.59 c**	**21.00 ± 1.12 ab**	**22.32 ± 0.63 a**	**17.95 ± 1.37 b**
Eriodictyol hexoside 1	2.24 ± 0.44 c	2.79 ± 0.53 c	11.90 ± 1.14 ab	14.00 ± 0.26 a	8.69 ± 0.95 b
Eriodictyol hexoside 2	1.75 ± 0.11 b	2.78 ± 0.60 b	8.05 ± 0.83 a	6.64 ± 0.33 a	7.66 ± 1.41 a
Naringenin hexoside	0.54 ± 0.01 c	0.54 ± 0.08 c	1.06 ± 0.04 b	1.68 ± 0.06 a	1.60 ± 0.07 a

It continues

Phenolic group	T1	T2	T3	T4	T5
**Flavonols**	**8.90 ± 0.25 c**	**10.37 ± 1.63 c**	**21.99 ± 1.00 b**	**30.67 ± 1.08 a**	**27.00 ± 1.35 ab**
Isorhamnetin-3-rutinoside	0.02 ± 0.003 b	0.04 ± 0.01 b	0.10 ± 0.008 a	0.13 ± 0.05 a	0.12 ± 0.01 a
Kaempferol-3-rutinoside	0.23 ± 0.04 b	0.58 ± 0.15 b	1.39 ± 0.11 a	1.76 ± 0.07 a	1.57 ± 0.13 a
Kaempferol acetyl hexoside	0.59 ± 0.06 b	0.63 ± 0.15 ab	0.922 ± 0.13 ab	1.04 ± 0.09 a	0.94 ± 0.03 ab
Kaempferol hexoside 1	0.34 ± 0.09 b	0.83 ± 0.17 b	1.62 ± 0.12 a	2.11 ± 0.09 a	1.75 ± 0.12 a
Kaempferol hexoside 2	0.29 ± 0.06 c	0.35 ± 0.08 c	1.04 ± 0.09 ab	1.26 ± 0.05 a	0.87 ± 0.11 b
Quercetin-3-galactoside	1.05 ± 0.08 c	1.23 ± 0.11 c	3.04 ± 0.29 b	3.93 ± 0.14 a	2.80 ± 0.11 b
Quercetin malonyl hexoside	0.10 ± 0.02 c	0.12 ± 0.03 c	0.37 ± 0.03 ab	0.45 ± 0.02 a	0.31 ± 0.04 b
Quercetin-3-glucoside	0.23 ± 0.04 d	0.38 ± 0.08 cd	1.18 ± 0.15 a	0.97 ± 0.04 ab	0.66 ± 0.08 bc
Quercetin-3- rutinoside	5.92 ± 0.14 c	5.99 ± 0.85 c	11.61 ± 0.38 b	18.44 ± 0.71 a	17.59 ± 0.80 a
Myricetin-3-glucuronide	0.14 ± 0.03 d	0.22 ± 0.05 cd	0.71 ± 0.09 a	0.58 ± 0.02 ab	0.39 ± 0.05 bc
**Total polyphenolics**	**48.15 ± 2.27 d**	**45.49 ± 4.03 d**	**168.63 ± 8.27 b**	**200.81 ± 5.22 a**	**121.14 ± 5.77 c**
