# Supplementary figures and images for: Identification of Phenolic Compounds in the Invasive Plants Staghorn Sumac and Himalayan Balsam: Impact of Time and Solvent on the Extraction of Phenolics and Extract Evaluation on Germination Inhibition
Source: Plants (Basel). 2024 Nov 28;13(23):3339. doi: 10.3390/plants13233339 (PMC11644323; doi:10.3390/plants13233339)

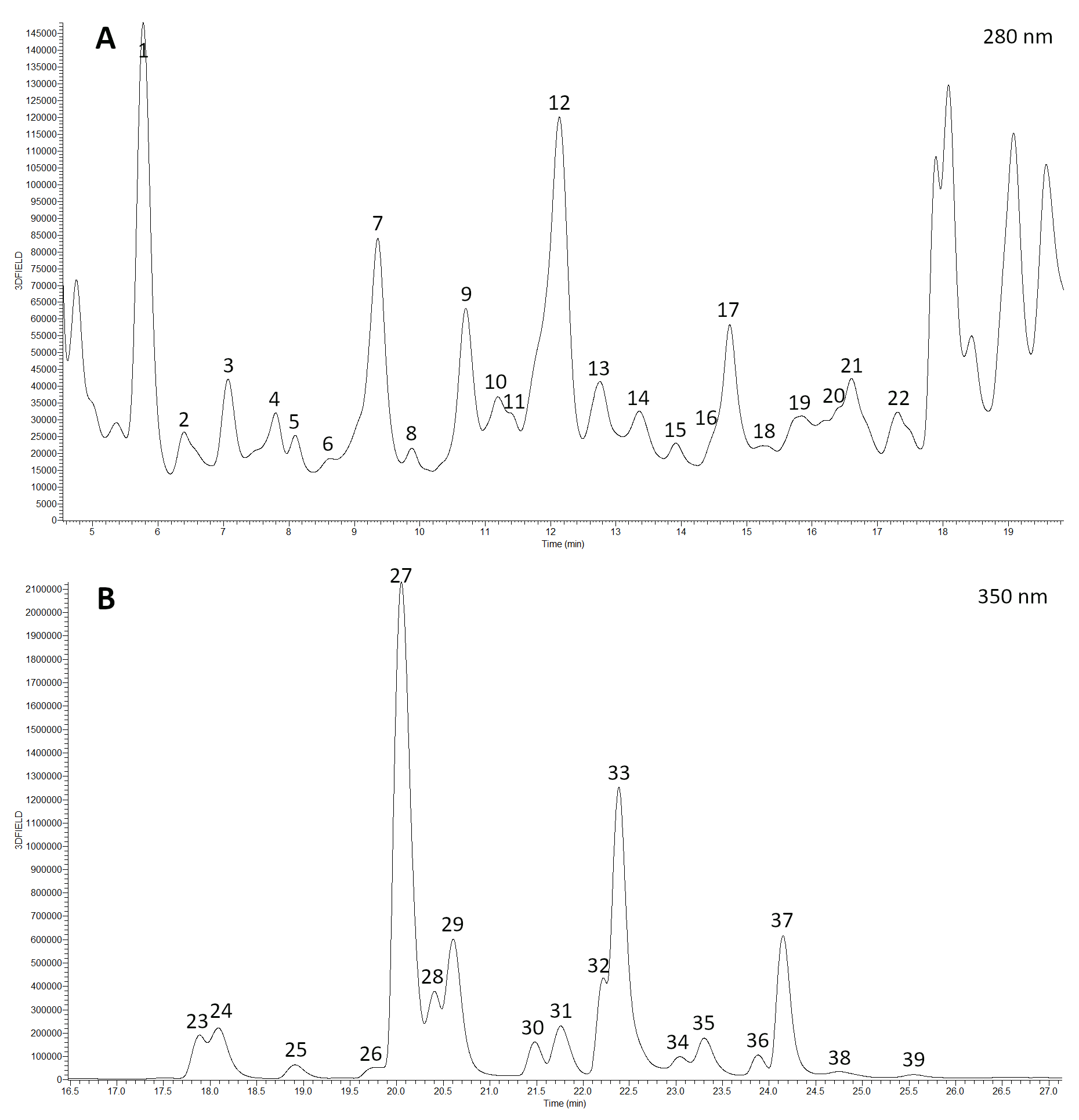

Supplement: Supplementary file 1 [file plants-13-03339-s001.zip › Supplementary Figure S1.png]

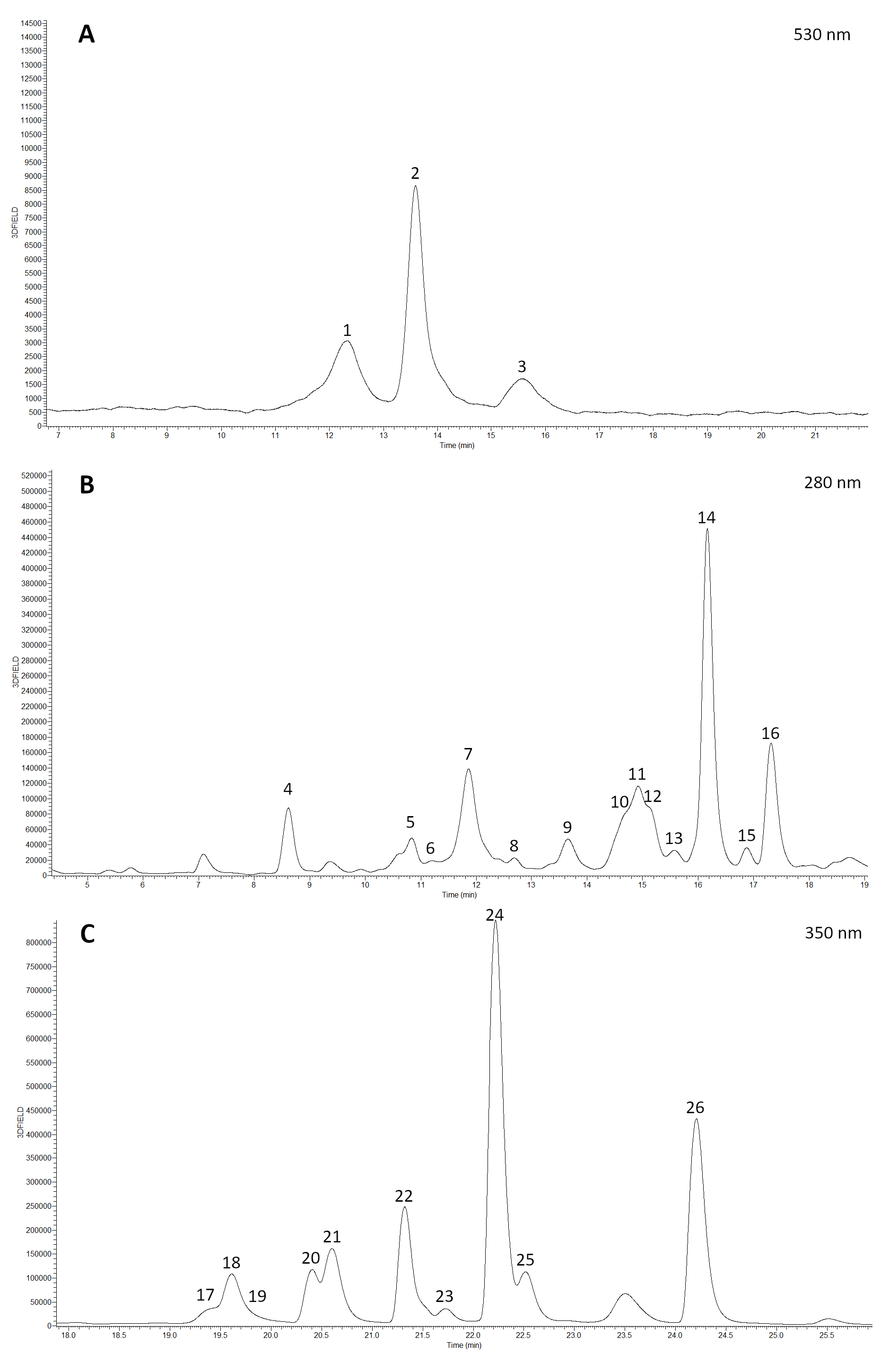

Supplement: Supplementary file 1 [file plants-13-03339-s001.zip › Supplementary Figure S2.png]
